# Assessment of tissue oxygenation of periodontal inflammation in patients with coronary artery diseases using optical spectroscopy

**DOI:** 10.1186/1472-6831-14-25

**Published:** 2014-03-25

**Authors:** Chunyang Zhang, Xiaoming Xiang, Minqi Xu, Chun Fan, Michael G Sowa, Kan-Zhi Liu

**Affiliations:** 1 The Affiliated Hospital of Medical College, Qingdao University, Qingdao, P.R. China; 2 Medical Devices Portfolio, National Research Council of Canada, 435 Ellice Ave., Winnipeg, MB R3B 1Y6, Canada; 3 Department of Dental Diagnostics and Surgical Sciences, University of Manitoba, Winnipeg, Canada

**Keywords:** Optical spectroscopy, Coronary artery diseases, Periodontitis, Tissue oxygenation

## Abstract

**Background:**

We have recently developed a non-invasive periodontal diagnostic tool that was validated in periodontitis patients without systemic disorders like coronary artery disease (CAD). The purpose of present study is to verify whether this optical instrument can also be used in periodontitis patients with CAD.

**Methods:**

A total of 62 periodontitis patients with CAD were recruited along with a control group consisting of 59 age and gender matched periodontitis volunteers without systemic disorders. Using a portable optical near-infrared spectrometer, optical spectra were obtained, processed and evaluated from the two groups. A modified Beer-Lambert unmixing model that incorporates a nonparametric scattering loss function was used to determine the relative contribution of deoxygenated hemoglobin (Hb) and oxygenated hemoglobin (HbO_2_) to the overall spectrum. The balance between tissue oxygen delivery and utilization in periodontal tissues was then assessed.

**Results:**

Tissue oxygen saturation was significantly decreased in the periodontitis sites (p < 0.01), compared to the healthy sites in those individuals with CAD. There was a trend towards increased concentration of Hb and decreased concentration of HbO_2_ from healthy to diseased sites, without statistical significance (p > 0.05). No statistical differences were found in tissue oxygen saturation between the CAD and control groups either in periodontal healthy or inflammatory sites.

**Conclusion:**

This study supports the hypothesis that optical spectroscopy can determine the periodontal inflammation in patients with certain systemic disorders like CAD. And the overall periodontal oxygenation profiles in CAD patients resemble those in non-CAD individuals either in healthy or inflammatory sites.

## Background

Increasing evidence has indicated that periodontitis, an endemic infectious disease of the tissues surrounding the teeth, may negatively impact systemic health. Particularly striking is the association between periodontitis and the increased risk of vascular diseases (including coronary artery disease and cerebral stroke) and diabetes mellitus [1,2].

Meta-analysis of data linking coronary artery diseases (CAD) and periodontitis suggests periodontitis is a significant risk factor for CAD in a dose-dependent manner, with relative risk ranging from 1.24 to 1.35 [3]. In addition to CAD, higher risk of cerebrovascular disease, such as stroke, was also found to be related to periodontal diseases [4]. It is also noted that many pediodontitic patients had increased carotid artery intimal medial thickness but reported no history of cardiovascular diseases, suggesting subclinical atherosclerosis was present in most periodontitis patients [5,6].

In spite of epidemiological evidence of significant association between established periodontitis and cardiovascular disease, the search for answers to why patients with periodontal disease are at higher risk for cardiovascular disease continues. Like other chronic inflammatory diseases, periodontitis has been associated with increased systemic inflammation, potentially leading to an increased risk of atherogenesis and other systemic disorders like diabetes and CAD [7,8].

Alternatively the local hemodynamic changes in periodontitis patients might be affected by the impact of atherosclerosis which in turn may manifest different clinical appearance. The pathological conditions of arteriosclerosis, hypertension or impaired heart function, often co-existing among cardiovascular diseases, disturb the systemic circulation and hence may have an influence on the gum microcirculation [5-8]. For instance, patients with carotid plaque measured by Echo-Doppler had higher periodontal indices demonstrated that the carotid peak wall shear stress was inversely related to all periodontal indices [9]. They proposed that the hemodynamic changes might contribute to atherosclerosis in periodontitis patients. However none has investigated whether the altered hemodynamic forces existing in atherosclerosis patient will affect diagnostic accuracy of periodontitis patients with CAD especially when a new diagnostic device is introduced.

Periodontal diseases are currently diagnosed almost entirely by their clinical manifestations, including attachment and bone loss, pocket formation, marginal bleeding, suppuration and bleeding on probing (BOP) [10]. Although clinical parameters are important tools to monitor healthy and diseased status and the response to treatments, they are not able to reliably identify susceptible individuals and distinguish active from inactive sites [11,12]. Thus, there are still major diagnostic and prognostic challenges for periodontal diseases. For this reason, our group and others have developed some novel non-subjective tools to supplement the current clinical diagnostic methods [13-15].

Recently, our research group has developed an optical device to improve the diagnosis of periodontal diseases. Although our previous data from multicenter studies [13,16,17] have demonstrated that optical spectroscopy is a very promising tool for the diagnosis of periodontal diseases, to date, this device has not been validated in periodontitis patients co-existing with other systemic disorders like CAD. Therefore, the first aim of this study is to validate this newly established periodontal diagnostic tool in periodontitis patients with CAD. And the second purpose is to document the local inflammatory hemodynamic profile (tissue oxygen saturation (StO_2_), total tissue hemoglobin (tHb), deoxyhemoglobin (Hb) and oxygenated hemoglobin (HbO_2_)) in those patients.

## Methods

### Study sites and subjects

The study was carried out at the Affiliated Hospital of Qingdao University (Qingdao, China) and the Second Affiliated Hospital of Harbin Medical University (Harbin, China). A total of 62 CAD patients (42 males, 20 females, with an age range of 51 to 71 years) with moderate to severe chronic periodontitis were recruited consecutively during the period of May to October 2012. Meanwhile, 59 systemically healthy patients (34 males, 25 females, with an age range of 33 to 58 years) with gingivitis and or periodontitis were also recruited and assigned to the control group for a comparative study. The research protocol was approved by each of the Research Ethics Boards of Affiliated Hospital of Qingdao University, the Second Affiliated Hospital of Harbin Medical University and the National Research Council of Canada. Informed, written consent was obtained from each individual before collection of spectra. CAD were diagnosed based on patient’s medical history, clinical symptoms, physical examinations and diagnostic tests such as electrocardiography, echocardiography, coronary computed tomography angiography (CCTA) or invasive coronary angiography (ICA), according to the diagnosis criteria recommended by the American College of Cardiology Foundation and the American Heart Association [18].

Periodontitis sites were defined as those with periodontal probing depth (PD) ≥ 5 mm, clinical attachment loss (CAL) ≥3 mm and bleeding on probing. Gingivitis sites were defined as those with PD < 3 mm and bleeding on probing. Healthy sites were defined as those with periodontal probing depths < 3 mm and no bleeding on probing [10]. Exclusion criteria were 1) tobacco smoking; 2) anti-inflammatory medications within the past three months (e.g. non-steroidal anti-inflammatory drugs, steroids, antibiotics, or immunosuppressants) that may interfere with the study; 3) certain systemic conditions that may interfere with the study, such as diabetes and immunological diseases; 4) use of orthodontic appliances; 5) pregnancy and lactation; 6) periodontal treatment within the past 12 months; 7) lesions of the gingival unrelated to plaque-induced periodontal disease; and 8) continuous use of mouthrinses containing antimibrobials with the past two months.

### Acquisition of optical spectra

Spectra were collected using a portable PDA512-ISA spectrograph interfaced to a customized bifurcated fiber optic probe designed for use in the oral cavity. The intraoral probe was described in detail previously [13]. The outer fibers of the probe were coupled to the entrance slit of the spectrograph and collected light subsequently back-scattered from the tissue. The inner fibers at the bifurcated end of the probe were coupled to a 5-watt tungsten halogen light source that provided a stable light output. Each reflectance spectrum consisted of 16 co-added scans collected using a 0.03 s integration time. The spectral range between 500 and 1100 nm at 5 nm resolution was used. A 99% Spectralon® reflectance standard was used as a reference to convert raw data into reflectance spectra. During the collection of the spectra, the participants were comfortably seated in a relaxed, standard semi-reclined position on a dental chair. Spectra were obtained from healthy, gingivitis and periodontitis sites from each eligible subject. A total of 890 (CAD: 379, control: 511) spectral measurements were acquired at sites of mid-facial, mid-lingual, mesio-facial, mesio-lingual, disto-facial or disto-lingual. All spectra were collected prior to clinical measurements and the spectral site distributions were shown in Table 1. All dental clinical parameters and optical spectra were assessed/obtained by two calibrated examiners (XMX & FC). We have assessed the variability generated from different operators and instruments by the matched pair experiments in the previous study [17]. No obvious difference was observed between the measurements made between users or within users.

**Table 1:** Distribution of spectral measurement sites

**Group**	**Diagnosis**	**Buccal**	**Lingual**	**Upper**	**Lower**	**MB/ML**	**IP**	**Total**
**Control**	Healthy	157	52	148	61	177	32	209
	Gingivitis	146	74	135	85	140	80	220
	Periodontitis	57	25	40	42	30	52	82
**CAD**	Healthy	142	29	96	75	88	83	171
	Gingivitis	127	28	71	84	42	113	155
	Periodontitis	37	16	31	22	5	48	53

MB = mid-buccal; ML = mid-lingual; IP = interproximal.

### Calculation of hemodynamic indices from optical spectra

The derivation of the relative contribution of Hb and HbO_2_ to the optical attenuation spectrum obtained from tissue was described in detail previously [19]. Briefly, a modified Beer-Lambert unmixing model that incorporates a nonparametric scattering loss function was used to determine the relative contribution of Hb and HbO_2_ to the spectrum by using the known absorption coefficients of Hb and HbO_2_ to fit the spectrum. The visible region between 510 and 620 nm of the measured tissue attenuation spectrum, Aλ, was modeled as a sum of two parametric terms, Hb and HbO_2_, that contribute to the spectrum and a nonparametric term m (λ) modeling a vector of covariates, primarily the Rayleigh and Mie scattering losses that contribute to the attenuation of measured light.

$$A\left(\lambda \right)=\sum_{i=1}^{3}\xi_{i}\left(\lambda \right)c_{i}L+m\left(\lambda \right)+\mathrm{error}$$

The concentrations of Hb and HbO_2_ per unit photon pathlength were estimated by solving an equation using a noniterative partially linear method based on kernel smoothing, as first described by Speckman [20]. StO_2_ and tHb, a measure of tissue perfusion, were derived from the predicted Hb and HbO_2_ relative concentrations as follows:

$$S_{t}O_{2}=\frac{\left[\mathrm{Hb}O_{2}\right]}{\left[\mathrm{Hb}O_{2}\right]+\left[\mathrm{Hb}\right]}\;\mathrm{and}\;\mathrm{tHb}=\left[\mathrm{Hb}O_{2}\right]+\left[\mathrm{Hb}\right]\;$$

### Statistical analysis

The hemodynamic indices, Hb, HbO_2_, StO_2_, and tHb, derived from optical spectral were analyzed separately using a one-way analysis of variance (ANOVA) to test the hypothesis that the indices from the three groups of sites (healthy, gingivitis and periodontitis) would differ significantly. The unequal Tukey HSD was used for the post-hoc pairwise comparisons of mean differences between the clinical groups. Pearson product moment correlation coefficients were calculated between the hemodynamic indices to summarize the linear association between the variables. Statistical calculations were performed with Statistica 7.1.

## Results

### Clinical parameters from the study group

Based on the periodontal probing depth, clinical attachment loss and dental presence as shown in Table 2, the most frequent type of periodontitis detected was of the chronic form. The mean probing depth for the periodontitis in non-CAD group was 6.38 ± 1.67 mm, while that of the gingivitis group was 2.19 ± 0.69 mm. Similarly, the PDs for the periodontitis in CAD group were 5.96 ± 1.73 mm and gingivitis group 2.50 ± 0.67 mm. Regarding the attachment loss, the mean was 7.39 ± 2.44 mm for the periodontitis in non-CAD group and 7.15 ± 2.65 mm in CAD group, with the upper molars being the most affected teeth.

**Table 2:** Clinical measurements from each group

**Group**	**Diagnosis**	**Clinical parameters**	
		**PD (mm)**	**CAL (mm)**
**Control**	Healthy (n = 209)	1.67 ± 0.65	2.16 ± 1.50
	Gingivitis (n = 220)	2.19 ± 0.69	2.83 ± 1.57
	Periodontitis (n = 82)	6.38 ± 1.67	7.39 ± 2.44
**CAD**	Healthy (n = 171)	2.01 ± 0.76	3.31 ± 1.62
	Gingivitis (n = 155)	2.50 ± 0.67	3.72 ± 1.46
	Periodontitis (n = 53)	5.96 ± 1.73	7.15 ± 2.65

### Hemodynamics extrapolated from optical spectra

**Figure 1:**
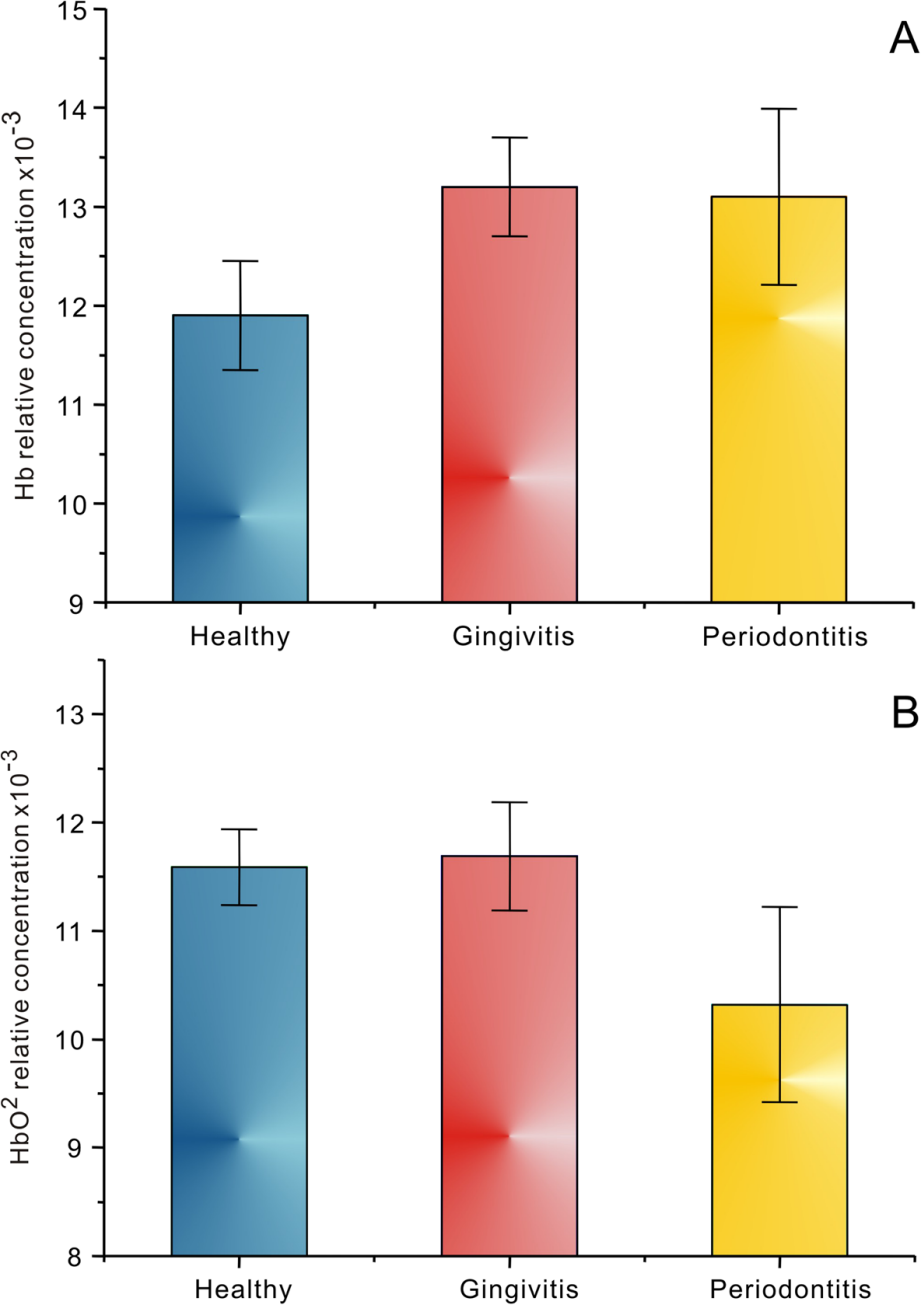
**Relative concentrations of Hb (A) and HbO**_**2 **_**(B) from healthy, gingivitis and periodontitis sites from CAD patients.** Relative hemoglobin concentrations were calculated using the visible region (510 – 620 nm) of the reflected light spectrum. Vertical bars denote 0.95 confidence intervals.

Figure 1A and B present the mean (± 95% CI) relative concentration of Hb and HbO_2_ obtained from the optical attenuation spectra of the healthy, gingivitis, and periodontitis sites of the CAD patients, respectively. Although the relative concentrations of Hb and HbO_2_ in the periodontal tissues of CAD patients were not significantly different among groups (p > 0.05), there was a trend towards increased mean concentration of Hb and decreased mean concentration of HbO_2_ from healthy to diseased sites.

**Figure 2:**
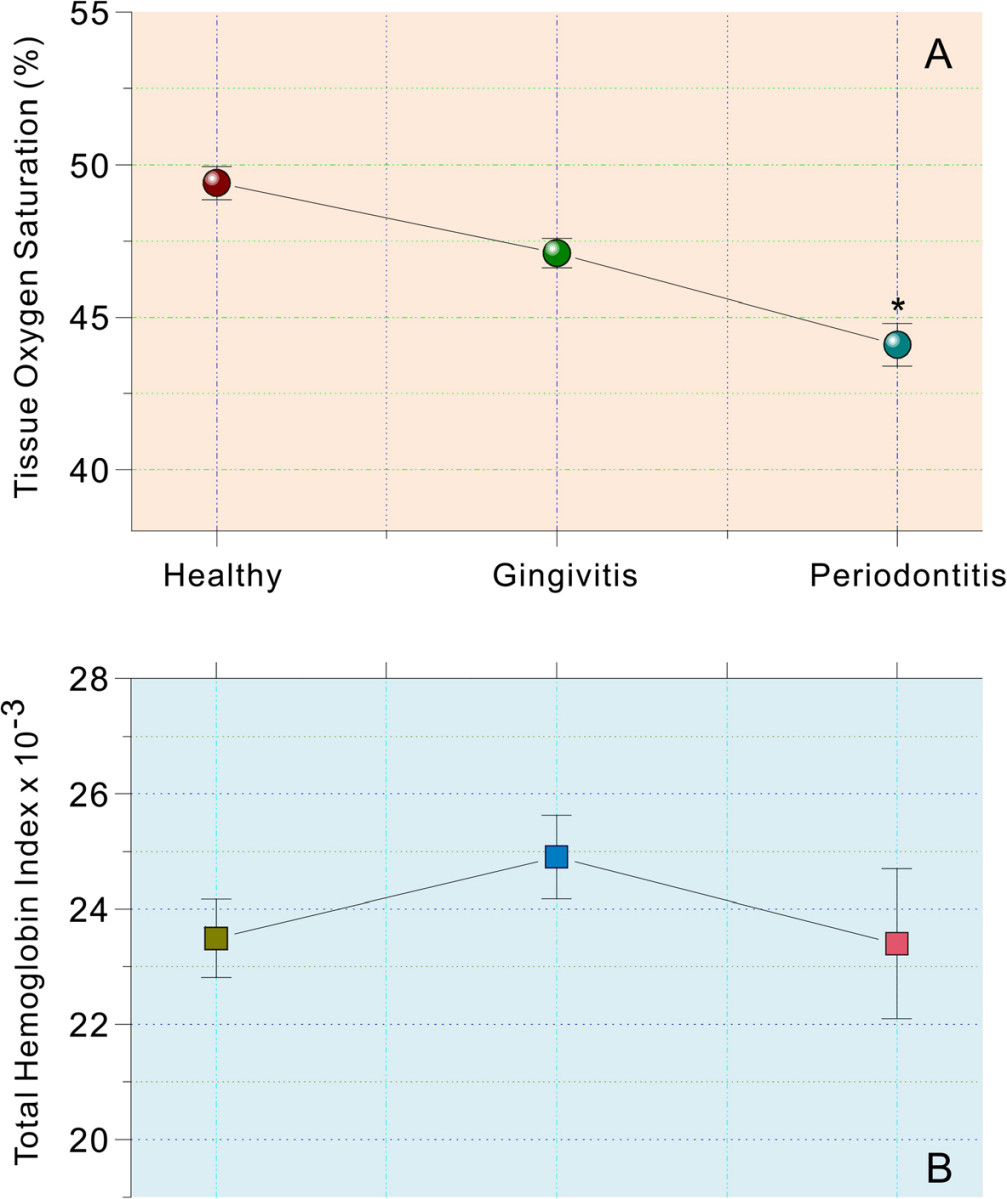
**% tissue hemoglobin oxygen saturation (A) and total Hb indices (B) derived from the relative concentrations of Hb and HbO**_**2**_**.** Indices were compared between healthy, gingivitis and periodontitis sites from CAD patients. *represents *P* < 0.001, compared with healthy group. Vertical bars denote 0.95 confidence intervals.

The spectral indices, StO_2_ and tHb in CAD group, that is closely related to oxygenation and blood volume of the tissue, are presented in Figure 2A and B, respectively. StO_2_ decreased from the healthy to the gingivitis (p = 0.07) or periodontitis (p = 0.002) status, while there was no significant difference between the gingivitis and periodontitis groups (p > 0.05). The profiles of tHb parameter, an indicator of tissue perfusion, did not differ among groups (all p > 0.60, Figure 2B).

**Figure 3:**
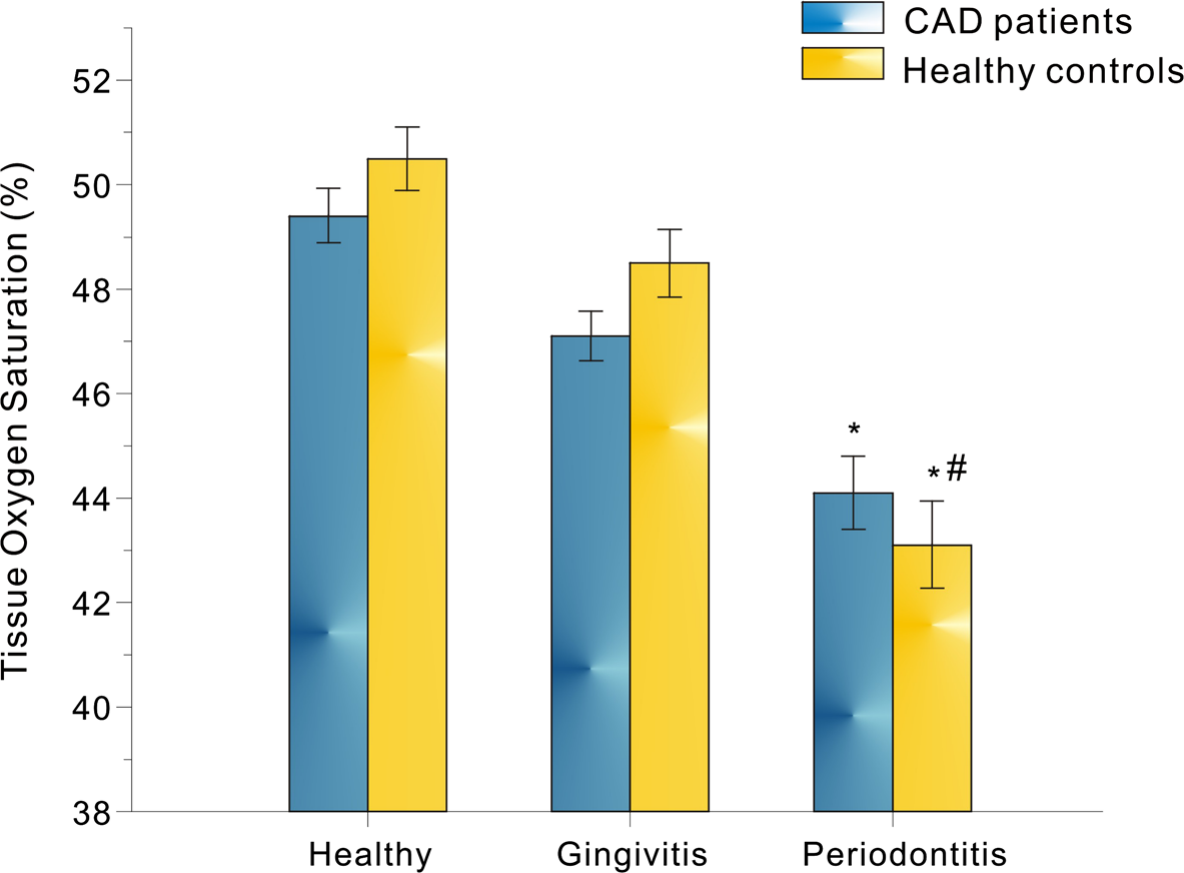
**Comparison of tissue oxygen saturations in percent from CAD patients and healthy controls.** *represents *P* < 0.01, compared with healthy group and #represents *P* < 0.05, compared with gingivitis group.

The StO_2_ in control and CAD groups were further compared in Figure 3. In control group, the StO_2_ decreased from the healthy to the gingivitis (p = 0.05) or periodontitis (p < 0.01) status, and there was significant difference between the gingivitis and periodontitis groups (p < 0.05). However, no significant differences were found when we compared the StO_2_ in control and CAD groups in all three pairs, i.e., healthy, gingivitis and periodontitis (p > 0.05).

## Discussion

The clinically-based diagnosis of periodontal diseases has been used for more than 50 years without significant improvements [21,22]. Although clinical parameters are important tools to monitor healthy and diseased status and the response to treatments, they are not able to reliably identify susceptible individuals and distinguish active from inactive sites [11,12]. Thus, there are still major diagnostic and prognostic challenges for periodontal diseases. For this reason, our group and others have developed some novel non-subjective tools to supplement the current clinical diagnostic methods [13-15]. In particular, we have developed a portable optical instrument using visible-near-infrared light to evaluate the local microcirculation of periodontal tissues by measuring tissue hemoglobin concentrations and oxygen saturation in a non-invasive and real-time manner [13,16,17,23]. Based on our current data bank generated from more than 400 participants, reflecting different ethnic backgrounds collected from six independent clinical centers, we have demonstrated that tissue oxygen saturation is significantly decreased in both gingivitis and periodontitis sites compared to healthy control sites. Interesting, this decrease is more pronounced in periodontitis than in gingivitis. Therefore, based on these hemodynamic parameters, our device can differentiate representative periodontal inflammatory conditions like gingivitis and periodontitis from healthy periodontal tissues.

However, in our previous studies we have intentionally excluded patients presenting risk factors or risk indicators (i.e. systemic disorders like diabetes mellitus, tobacco smoking and CAD) for periodontitis that may affect overall spectral outcome [13,16,17,23]. At this phase of our optical instrument validation, supplementary studies are needed to address whether this technology can be also useful for patients presenting systemic diseases like CAD, without major undesirable influence. Therefore, the primary purpose of this study was to monitor with optical spectroscopy the local gingival oxygenation in healthy and diseased periodontal sites of CAD patients.

As demonstrated in this report, the spectral profile of periodontal sites in CAD patients generally resembles those observed in our previous multicenter studies on non-CAD systemically healthy patients [13,17,23]. For example, StO_2_ was significantly lower in the inflamed compare to the healthy gingival sites (Figure 2A). Such decreased StO_2_ probably reflects tissue hypoxia resulting from an ongoing inflammatory response in the periodontal tissues, which leads to increased oxygen consumption [24]. It is well known that, in destructive periodontal diseases, anaerobic microorganisms predominate in the periodontal pockets as diminished oxygen tension in the bottom of the pockets would be prompt for the growth of anaerobic bacteria [25,26]. Meanwhile, the altered subgingival bacterial profile in the periodontitis of CAD may also contribute to the reduced level of StO_2_ observed in the present study. For instance, it has been revealed that there is an association between subgingival levels of etiological bacteria for periodontitis and carotid artery intima-media thickness, and the presence of CAD, respectively [27,28]. In addition, periodontal pathogen burden in subgingival biofilm samples has also been associated with carotid intima-media thickness and with increased odds for myocardial infarction and CAD [27,29]. Alternatively, the pocket depth has been indicated to may also play an important role in tissue oxygen saturation. It has found that tissue oxygen saturation correlated well with oxygen tension in periodontal pockets since oxygen tension tends to decline as pocket depth increases [30]. This was further confirmed in this study that the average pocket depths of control, gingivitis and periodontitis in CAD group are increased from 2.01 mm to 2.50 mm and to 5.96 mm and along with corresponding tissue oxygen saturation decrease (Table 2, Figure 2).

Until now, study of the periodontitis and cardiovascular disease relationship has primarily concentrated on clinical cardiovascular events, while recently some studies inclined to investigate the relationship of periodontitis to subclinical measures of atherosclerosis. For instance, the intima-media wall thickness (IMT) of the carotid artery, as measured by B-mode ultrasound, is a measure of preclinical atherosclerosis that has been shown to be associated with CAD, both prevalent [31] and incident [32,33], and with incident stroke [34,35]. In particular, an early study by Beck et al has demonstrated that periodontal disease was associated with IMT of the carotid arteries, with abnormal thickening significantly more prevalent among subjects with severe periodontitis [36]. A recent intervention study further indicated that periodontal therapy decreased total oral bacterial load, inflammation biomarkers, adhesion and activation proteins and carotid IMT [37]. By examining the hemodynamic profile of wall shear stress in carotid artery, Carallo et al directly investigated the link between periodontal inflammation and atherosclerosis, and their data suggested that local hemodynamic changes might contribute to atherosclerosis in periodontitis patients [9].

However, none has looked into whether the altered hemodynamic forces existing in atherosclerosis patient will directly affect the local tissue oxygenation of periodontitis patients with CAD due to the limitation of available techniques. To our best knowledge, current study was the first report to document the local inflammatory hemodynamic profile in CAD patients with periodontal disease. As demonstrated in Figure 3, there are no statistical differences between CAD and non-CAD control groups in term of their tissue oxygenations, regardless it in healthy or periodontal inflammatory sites. Although the precise mechanisms remain to be clarified, the current observation would suggest that the local oxygenation status in periodontal tissue was not affected by CAD status in spite of other preclinical atherosclerosis or related hemodynamic changes existing in local or adjacent circulation like carotid artery [9,36,37].

Nevertheless, it is important to note that some CAD-associated masking effects on periodontal inflammation could be observed by the optical spectra in the present study. For instance, although there was a trend towards lower level of StO_2_ in periodontitis than gingivitis sites in CAD patients, this difference was not statistically supported (P = 0.056) (Figure 3). In contrast the StO_2_ levels in periodontitis sites in Non-CAD was significantly decreased comparing to that of gingivitis (P < 0.05) similar to our previous reports [13,17]. Again the precise mechanism remains to be explored. Given the fact that angiopathies such as arthrosclerosis and arteriostenosis are common in CAD, it cannot be ruled out that CAD associated hemodynamic changes might have some relationship with those angiopathies which may further disturb periodontal microcirculation and cause gum-inflammation-induced hemodynamic shifting. Therefore, it is highly likely that one of the possibilities responsible for the so called CAD associated “masking effect” may be attributed to the definite atherosclerosis and/or CAD actions on gingival vessels and/or aggregated periodontal inflammations in CAD patients [9,36].

In summary, the current study suggests that pathologic conditions like CAD will not affect our newly established periodontal diagnostic tool when used on CAD patients with periodontitis. Alternatively due to the fact that tissue oxygen saturation is not measureable clinically, our data would indicate that optical spectroscopy can serve as a supplementary tool for monitoring local oxygenation and provide essential information about local inflammation even in patients with CAD. Furthermore, since periodontal hemodynamic indices such as tissue oxygenation and perfusion are not clinically measurable, the development and validation of sensitive and specific tools to reveal the characteristics of periodontal hemodynamics in CAD patients have direct and relevant clinical implications. It is important to point out that the practical implications of the optical spectroscopy reported herein may be beyond the diagnosis of periodontitis with CAD. It may potentially serve as an alternative biologically-based assessment of periodontal inflammation. Future studies are warranted to evaluate other risk factors or risk indicators (i.e. systemic disorder like diabetes mellitus and among smokers) for periodontitis that may affect overall spectral outcome. Prospective longitudinal studies are also warranted to test this tool in differentiating progressive from stable lesions in monitoring periodontal inflammation in connection to the long-term stability of periodontal tissue.

## Conclusions

In summary, our data demonstrated that the visible-infrared spectral device can be a sensitive means to monitor the local periodontal inflammation with or without other systemic disorders like CAD disease by assessing the periodontal hemodynamic profiles. This novel tool will also allow scientists to better explore the essential relationship between periodontal disease and CAD. Future parallel cohort studies with expanded subject size are warranted to establish a large data base to understand the influence of other systemic disorders on periodontal inflammations embedded in optical spectra.

## Abbreviations

CAD: Coronary artery diseases; Hb: Deoxygenated hemoglobin; HbO2: Oxygenated hemoglobin; PD: Probing depth; CAL: Clinical attachment loss; StO2: Tissue oxygen saturation; tHb: Total tissue hemoglobin; CCTA: Coronary computed tomography angiography; ICA: Invasive coronary angiography; IMT: Intima-media wall thickness.

## Competing interests

The authors report no conflicts of interest related to this study.

## Authors’ contributions

The original hypothesis was developed by CYZ and KZL. CYZ and CF identified and recruited suitable clinical cases. XMX, MQX and CF performed periodontal assessments and spectral data acquisitions. MQX and MGS performed the extrapolation of data from the IR spectra and the spectral data analysis. MQX and KZL drafted the manuscript. KZL and MGS obtained the funding. All authors read and approved the final manuscript.

## Pre-publication history

The pre-publication history for this paper can be accessed here:

http://www.biomedcentral.com/1472-6831/14/25/prepub
